# Direct Isomaltulose Synthesis From Beet Molasses by Immobilized Sucrose Isomerase

**DOI:** 10.3389/fbioe.2021.691547

**Published:** 2021-07-16

**Authors:** Qin-Qing Wang, Ming Yang, Jian-Hua Hao, Zai-Chao Ma

**Affiliations:** ^1^National Glycoengineering Research Center, State Key Laboratory of Microbial Technology, Shandong University, Qingdao, China; ^2^School of Marine Sciences, Sun Yat-sen University, Guangzhou, China; ^3^Helmholtz International Lab for Anti-Infectives, Shandong University-Helmholtz Institute of Biotechnology, State Key Laboratory of Microbial Technology, Shandong University, Qingdao, China; ^4^Key Laboratory of Sustainable Development of Polar Fishery, Ministry of Agriculture and Rural Affairs, Yellow Sea Fisheries Research Institute, Chinese Academy of Fishery Sciences, Qingdao, China; ^5^Laboratory for Marine Drugs and Bioproducts, Qingdao National Laboratory for Marine Science and Technology, Qingdao, China

**Keywords:** beet molasses, isomaltulose, sucrose isomerase, immobilization, economic analysis

## Abstract

Isomaltulose is becoming a focus as a functional sweetener for sucrose substitutes; however, isomaltulose production using sucrose as the substrate is not economical. Low-cost feedstocks are needed for their production. In this study, beet molasses (BM) was introduced as the substrate to produce isomaltulose for the first time. Immobilized sucrose isomerase (SIase) was proved as the most efficient biocatalyst for isomaltulose synthesis from sulfuric acid (H_2_SO_4_) pretreated BM followed by centrifugation for the removal of insoluble matters and reducing viscosity. The effect of different factors on isomaltulose production is investigated. The isomaltulose still achieved a high concentration of 446.4 ± 5.5 g/L (purity of 85.8%) with a yield of 0.94 ± 0.02 g/g under the best conditions (800 g/L pretreated BM, 15 U immobilized SIase/g dosage, 40°C, pH of 5.5, and 10 h) in the eighth batch. Immobilized SIase used in repeated batch reaction showed good reusability to convert pretreated BM into isomaltulose since the sucrose conversion rate remained 97.5% in the same batch and even above 94% after 11 batches. Significant cost reduction of feedstock costs was also confirmed by economic analysis. The findings indicated that this two-step process to produce isomaltulose using low-cost BM and immobilized SIase is feasible. This process has the potential to be effective and promising for industrial production and application of isomaltulose as a functional sweetener for sucrose substitute.

## Introduction

Molasses is a viscous by-product of sugar refineries with a sweet taste and color ranging from brown to dark-brown, which mainly includes cane molasses (CM), soy molasses (SM), and beet molasses (BM). The molasses is rich in sucrose and is also composed of a minimum amount of carbohydrates (like glucose) and other components (e.g., proteins, vitamins, and heavy metals) (Wang et al., 2019a; Palmonari et al., 2020). Particularly, large amounts of BM, annually, (more than 300,000 tons) were discharged in China in the past years, and only a little amount was used as grinding aid, feed, and carbon source (Gao et al., 2011; Yan et al., 2011), aggravating BM waste and causing serious environmental concerns. In other countries, BM has been utilized as sucrose-containing feedstock for microbes to produce value-added products, such as bioethanol (Razmovski and Vučurović, 2012; Vučurović et al., 2019), lipids (Taskin et al., 2016), hydrogen (Emrah et al., 2018), baker’s yeast (Ferrari et al., 2001), and inulinase (Germe and Turhan, 2020). Therefore, BM is a very valuable raw material. Recently, BM has attracted increasing attention for different industrial applications worldwide, including China, due to its higher sucrose content (about 50%) than those (about 30–50%) of other molasses (Álvarez-Cao et al., 2019; Ozdal and Başaran Kurbanoglu, 2019; Wang et al., 2019a; Zheng et al., 2019; Palmonari et al., 2020; Zhan et al., 2020).

Sucrose is of importance as a widely used sweetener in the daily diet; however, high sucrose diet could trigger health problems, such as obesity, dental caries, type 2 diabetes, and Alzheimer’s disease, due to the rapid increase in blood glucose and high sweet content of sucrose (Tian et al., 2019; Du et al., 2020; Yeh et al., 2020). Therefore, safe sweeteners with suitable sweetness were required for sucrose alternatives. Isomaltulose shows a similar appearance and taste to sucrose (Zhang et al., 2020) and has approximately 50% sweetening power of sucrose (Shyam et al., 2018). In addition, isomaltulose has been authorized as “generally recognized as safe” by the United States Food and Drug Administration (Mu et al., 2014), sharing advantageous health benefits including lower glycemic index, lower calorie content, body-weight reduction, higher stability, more tooth-friendly, and promoting probiotics as well as prebiotics activities (Shyam et al., 2018; Lightowler et al., 2019; Wang et al., 2019a; Lee et al., 2020). Hence, isomaltulose is a promising functional sweetener and can be utilized as an available sucrose substitute.

As a structural isomer of sucrose, isomaltulose is a natural disaccharide consisting of glucose and fructose connected by α-1,6-glycosidic bond. It can be converted from sucrose through isomerization catalyzed by sucrose isomerase (SIase), which is primarily derived from microbial processes and also produces small amounts of trehalulose, glucose, and fructose (Liu et al., 2020; Zhang et al., 2020). SIase-producing microbes for isomaltulose biosynthesis have been mainly found in bacteria including *Pantoea dispersa* (Wu and Birch, 2004), *Enterobacter* spp. (Park et al., 2010), *Protaminobacter rubrum* (de Oliva-Neto and Menão, 2009), *Serratia plymuthica* (Duan et al., 2016), *Klebsiella* spp. (Li et al., 2003), and *Erwinia* spp. (Li et al., 2011). Recently, to improve isomaltulose production *via* a safe and efficient enzymatic method, various measures on heterologous expression of SIase gene, such as optimizing the promoter and/or signal peptide (Park et al., 2010; Zhang et al., 2018), site-directed mutagenesis for high thermostability (Duan et al., 2016), and displaying it on the cell surface of bacteria and fungi (Lee et al., 2011; Li et al., 2013, 2017; Wu et al., 2017; Zheng et al., 2019; Zhan et al., 2020), are explored. Among the targeted expression systems above, an ascomycetous yeast, *Yarrowia lipolytica*, seems to be the most attractive one based on relatively high isomaltulose yield (0.96 g/g) production (Li et al., 2017; Zhang et al., 2018). Noteworthily, sucrose substrate is indeed responsible for cell growth as a carbon source and isomerization of SIase during bioconversion processes, leading to the decrease of isomaltulose yield and unexpected products formation (Li et al., 2017; Zhang et al., 2018; Tian et al., 2019). The inner consumption of sucrose and non-target products formation are still inevitable, including using free or immobilized cells for isomaltulose production, suggesting that using free or immobilized SIase to produce isomaltulose directly will be expected to reduce the formation of non-target products (Li et al., 2003, 2011; Tian et al., 2019).

The cost of sucrose for isomaltulose production accounts for more than half of the total cost (Wang et al., 2019b). To reduce the cost of isomaltulose production for wide industrial applications, using cost-effective feedstocks as substrate apart from efforts in improving SIase expression is also needed. BM is a low-cost and readily available raw material, which has been employed as a carbon source for microbial growth; however, a strategy to use BM as a simple substrate for isomaltulose production has not been reported yet.

Several engineered *Y. lipolytica* strains with food grade can be used to produce isomaltulose from sucrose, CM, and SM (Zhang et al., 2018; Wang et al., 2019a,b). Pretreated SM was exactly used as a favorable substrate to produce isomaltulose by microbial fermentation (Wang et al., 2019b), and immobilized SIase could elevate the conversion rate of sucrose and isomaltulose concentration (Zhang et al., 2019). This study aims to explore the pretreatment and the effect of different factors on the potential of low-cost BM as the sole substrate for isomaltulose production using immobilized SIase. The final goal of this investigation is to reduce enzyme demand and explore the feasibility of this process for the industrial production of isomaltulose by economic analysis.

## Materials and Methods

### Strain and Media

The yeast *Y. lipolytica* JD is a transformant carrying the strong promoter (TEFin) and SIase gene from *P. dispersa* UQ68J, which was cultivated in the medium containing glucose (30 g/L) and corn steep powder (20 g/L) with an initial pH of 6.0 for 72 h under the conditions of 30°C and 180 rpm to achieve extracellular SIase (49.3 U/mL) (Zhang et al., 2019). The strain JD was used for SIase production in this study. Another strain, *Y. lipolytica* G82, expressing the α-galactosidase gene was used to produce extracellular α-galactosidase (121.6 U/mL) when cultivated in the GPPB medium [containing 30 g/L glucose, 1.0 g/L (NH_4_)_2_SO_4_, 6.0 g/L yeast extract, 2.0 g/L KH_2_PO4, 3.0 g/L K_2_HPO_4_, and 0.1 g/L MgSO_4_.7H_2_O, pH 6.0] at 30°C for 3 days (Wang et al., 2019b).

### Determination of Sugar Concentration of Molasses

Raw CM was obtained from a sugar refinery in Guangxi. Raw SM was provided from a local soybean oil factory, and raw BM was supplied from Xinjiang. Different sugars contents of these raw molasses were detected by high-performance liquid chromatography (HPLC) using an Agilent 1200 system (Agilent Technologies, United States) and NH_2_ column (Thermo Fisher Scientific, United States). The carbohydrates (e.g., sucrose, glucose, fructose, stachyose, xylose, galactose, isomaltulose, and trehalulose) were all calculated according to peak areas and retention time.

### Pretreatment of BM

Beet molasses was treated as the described method (Cheng et al., 2017). Briefly, BM was acidified to the pH of 3 by 3 M sulfuric acid (H_2_SO_4_) and boiled for 5 min. The mixture would be centrifuged at 8,000 rpm for 30 min after keeping it at 4°C for about 12 h. Ca(OH)_2_ was added to the supernatant obtained to adjust pH to 6. Then, it was centrifuged at the same condition to remove insoluble matters and evaporated to obtain the dry matter as 78.0%. Then, the pretreated BM was diluted with distilled water to the targeted content. The clarified molasses solution was stored at 4°C until further use.

### SM Pretreated by α-Galactosidase

The pretreatment of SM was carried out by adding α-galactosidase with the activity of 121.6 U/mL from the crude fermentation broth of G82 strain, which was cultivated at 45°C and pH of 4.5 for 4 h, and the usage of α-galactosidase was 15 U/g of SM as described by Wang et al. (2019b). For sugar content determination, the obtained hydrolysate was analyzed by HPLC.

### Isomaltulose Production Using Free and Immobilized SIase

Isomaltulose production was conducted by free SIase and immobilized SIase from the crude fermentation broth of *Y. lipolytica* strain JD using sucrose as substrate, BM, and pretreated BM. Crude-free SIase was obtained by centrifugation (10,000 × *g*, 20 min, 4°C). In addition, SIase immobilization was carried out according to the previous study (Zhang et al., 2019). Briefly, polyvinyl alcohol (PVA, 10%) and sodium alginate (1%) were mixed with distilled water to obtain the final concentration of 1 g/100 ml and autoclaved at 115°C for 20 min. Then, SIase solution (40 U/g) was dropped to the PVA-alginate mixture after cooling it to room temperature. The SIase-PVA-alginate suspension (2 ml) was added through syringe needle dropwise into the crosslink boric acid solution (pH 8.0) under continuous steering, and the beads of PVA-borate SIase were immersed in it for 2 h at 4°C to ensure sufficient gelation reaction. Subsequently, the beads were washed with distilled water to remove the excess borate ions from the surface. The enzyme recovery rate was defined as the ratio of immobilized SIase activity to total SIase activity. Free or immobilized SIase was added to 300 g/L sucrose or 500 g/L BM, with the SIase dosage of 15 U/g at 40°C and pH 6 in the isomerization process. The sucrose concentrations in 500 g/L raw BM and pretreated BM were 256 g/L and 304.5 g/L, respectively, making the sucrose contents similar to 300 g/L. The produced isomaltulose content was also analyzed by using HPLC.

### Investigation of the Effect of Main Process Parameters on the Conversion of Pretreated BM to Isomaltulose

The catalysis condition of immobilized SIase was determined for the best of isomaltulose production using one-way ANOVA. The reaction factors pH (5.5–7.5), pretreated BM of different concentrations (400–900 g/L), and SIase adding dosages (5–25 U/g) were implemented for isomaltulose production. Corresponding sucrose concentrations in pretreated BM were 243.6 g/L, 304.5 g/L, 365.4 g/L, 426.3 g/L, 487.2 g/L, and 548.1 g/L, respectively. The reusability of immobilized SIase was evaluated under the best conditions for 16 cycles based on the conversion rate of the substrate at 40°C and pH 6.0. When the optimal duration of 12 h of each cycle was finished, the beads were filtrated with a 1 μm filter membrane and washed with distilled water, and then reused in the next cycle (Zhang et al., 2019). The contents of isomaltulose and others in the mixture in each cycle were determined by HPLC.

### Process Parameters

Related parameters in this study were all calculated. Isomaltulose proportion (%) was calculated with the content of produced isomaltulose divided by total sugars containing other produced sugars and residual sugars. Sucrose proportion (%) was presented with the sucrose content divided by the content of molasses. The yield of isomaltulose using immobilized or free SIase (g/g) represents the isomaltulose content divided by the sucrose content in molasses. Sucrose conversion rate (%) was described as the consumed sucrose for isomaltulose production divided by the total sucrose content in molasses. The dosage of SIase (U/g) was calculated with enzyme activity (U) divided by sucrose content (g). The dosage of α-galactosidase (U/g) meant that enzyme activity (U) was divided by SM content (g).

### Statistical Analysis

All tests were performed three times. The obtained data in experiments were analyzed through one-way ANOVA using SPSS 22.0 software (SPSS Inc., Chicago, MI, United States), and showed as mean ± SD. *p-*values were calculated by Student’s *t*-test (*n* = 3), and considered statistically significant when *P* < 0.05.

## Results and Discussion

### Difference of Sugar Content in Three Kinds of Molasses

**FIGURE 1:**
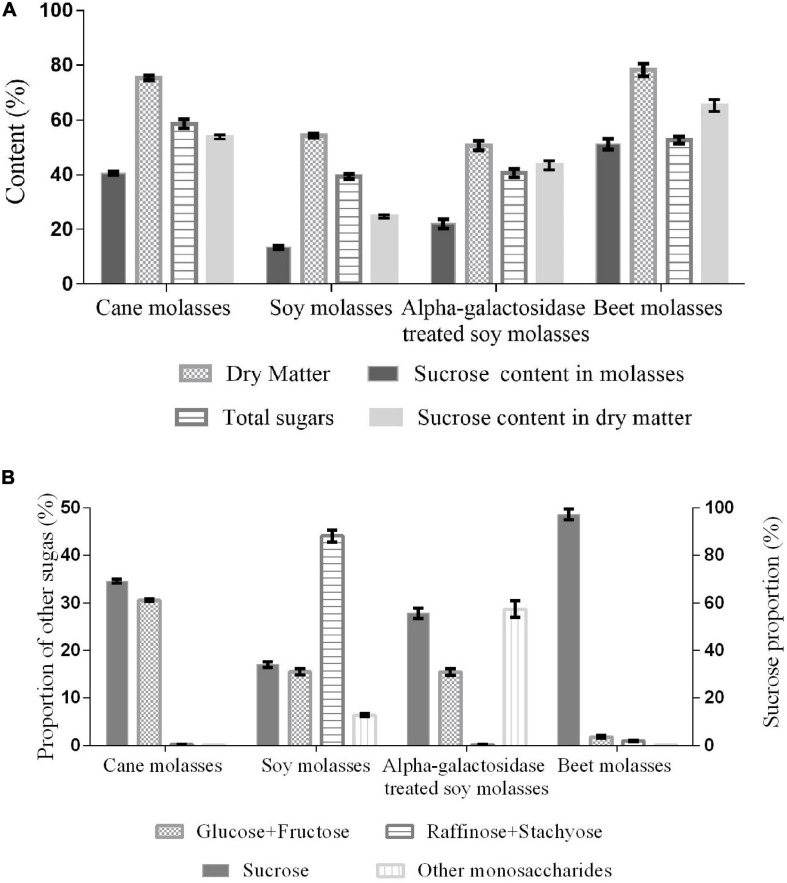
Sugar content **(A)** and proportion **(B)** in different molasses.

Molasses as low-cost feedstock plays an important role in microbial production due to high sugar content (Zhan et al., 2020). The sucrose content is responsible for the performance to be the substrate for producing isomaltulose. The sucrose content (51.2%) and dry matter (78.3%) in BM were shown in Figure 1A, the sucrose proportion reached 97.2% in all the carbohydrates of BM, which is the highest among the molasses, including α-galactosidase treated SM. In addition to the major constituent sucrose, SM and CM had a higher content of other sugars. For instance, approximately 30% of monosaccharides (glucose and fructose) in CM and 44% of raffinose family oligosaccharides in SM were also exhibited (Figures 1A,B). This phenomenon possibly results from the differences in raw materials and processing technology of sugar manufacturing (Álvarez-Cao et al., 2019; Palmonari et al., 2020).

Feeding livestock with molasses is likely to bring metabolic diseases (urea toxicity, molasses toxicity, and bloat) (Senthilkumar et al., 2016). Microorganisms can use the sugars of molasses both as a carbon source and substrate through fermentation of food-grade strains to generate diverse high value-added products, such as bioethanol, α-galactosidase, and isomaltulose (Álvarez-Cao et al., 2019; Roukas and Kotzekidou, 2020; Zhan et al., 2020). For example, CM (350 g/L) could be used to produce 161.2 g/L isomaltulose by *Y. lipolytica* S47 strain (Wang et al., 2019a). By contrast, BM richer in sucrose than CM would show a higher capacity to serve as a suitable cost-effective substrate for isomaltulose production.

### Comparison of Isomaltulose Production Using Free and Immobilized SIase

**FIGURE 2:**
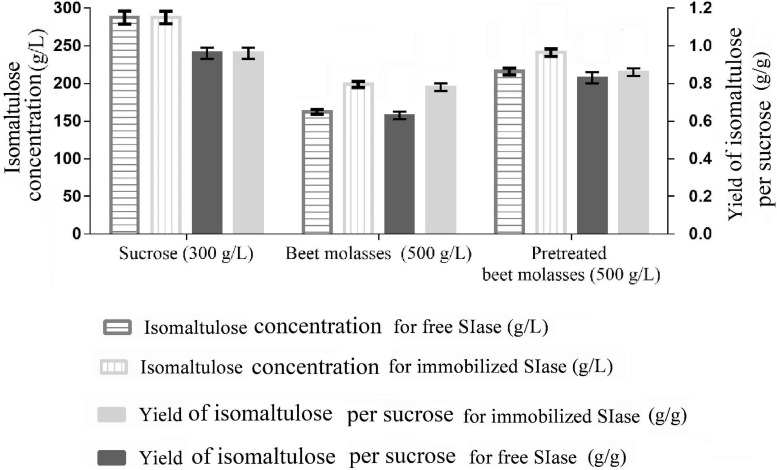
Isomaltulose production from different substrates by free and immobilized sucrose isomerase (SIase) under the conditions of SIase dosage of 15 U/g, pH of 6, and temperature of 40°C.

To avoid sucrose consumption and non-target products formation, specific enzyme catalysis application instead of free or immobilized microbial cells has been approved, which could improve substrate concentration and product purity (Zhang et al., 2019). As shown in Figure 2, the productions (287.8 ± 8.3 g/L) of isomaltulose synthesized by free SIase and immobilized SIase from sucrose were of the same highest values, and so did the yields (0.96 ± 0.03 g/g), among all the substrates. The results obtained keep the highest level from engineered *Y. lipolytica* strains using sucrose or CM to date (Zhang et al., 2018; Wang et al., 2019a).

Using immobilized SIase, the substrate pretreated BM achieved a higher isomaltulose production (240.8 g/L) and a higher yield (0.86 g/g) compared with free SIase, which were all significantly higher than those (198.8 g/L, 0.78 g/g) from raw BM using free or immobilized SIases (Figure 2). In contrast, the results obtained from raw BM decreased possibly due to the existence of metal ions restraining SIase activity. Most isomerases, including SIase, have been proved to be inhibited by metal ions, such as Ca^2+^, Ba^2+^, Cu^2+^, Zn^2+^, which are relatively rich in BM (Palmonari et al., 2020; Zhang et al., 2020). Pretreatment can reduce the content of those metal ions in BM, thus increasing the isomaltulose yield.

However, the production and yield of isomaltulose obtained from pretreated BM using immobilized SIase still hold distinctly higher levels than those catalyzed by different immobilized cells (such as *Erwinia* sp. D12, *Klebsiella* sp. K18, and *S. plymuthica* ATCC15928) (Mu et al., 2014) and many recombinant strains like *Escherichia coli* BL21(DE3) and *Y. lipolytica* (Li et al., 2017; Zheng et al., 2019; Zhang et al., 2020). The results confirmed that the pretreatment of BM is essential for efficient isomaltulose production. Simultaneously, immobilization of SIase benefits for production and yield of isomaltulose from pretreated BM.

### The Effect of Main Process Parameters on Isomaltulose Production by Immobilized SIase

**FIGURE 3:**
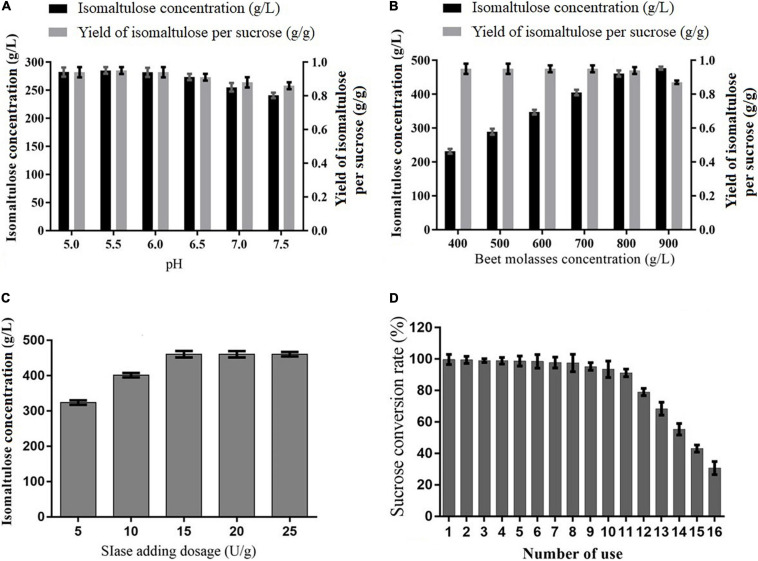
Effects of different factors on isomaltulose production from pretreated beet molasses (BM) by immobilized SIase. **(A)** pH, **(B)** pretreated BM concentration, **(C)** SIase adding dosage, and **(D)** the effect of recycling number of immobilized SIase on sucrose conversion rate.

To further enhance the catalysis of immobilized SIase forisomaltulose synthesis, the catalysis conditions were examined. As exhibited in Figure 3A, isomaltulose was produced efficiently in a wide range of pH (5–7.5), especially ranging from 5 to 6, and the pH 5.5 was most suitable for isomaltulose synthesis, showing that the process is pH-dependent and agrees with the activity of immobilized SIase (Zhang et al., 2019). In general, high substrate concentration facilitates catalysis using the immobilized enzyme. It was observed that the production of isomaltulose was continually rising as pretreated BM concentration increased, while the yield showed no significant changes before 800 g/L pretreated BM but reduced remarkably beyond it (Figure 3B), achieving the best pretreated BM concentration of 800 g/L, containing 487.2 g/L of sucrose. The high concentration of pretreated BM could be used for isomaltulose production due to the good tolerance of the immobilized SIase to high sucrose concentration (700 g/L) (Zhang et al., 2019), and the H_2_SO_4_-based pretreatment removing insoluble matters and reducing the viscosity of BM (Cheng et al., 2017); however, the concentration of 900 g/L pretreated BM, equivalently with 668.7 g/L whole dry matter, was not dissolved very well, indeed, which would be the main point responsible for lowering the yield of isomaltulose. This finding is higher than 350 g/L CM but is similar to the reported 800 g/L SM used to produce isomaltulose (Wang et al., 2019a,b). Besides, the results in Figure 3C implied that 15 U/g of SIase was the best dosage from a series of dosages examined for isomaltulose production.

The reusability of immobilized SIase also needed to be evaluated in terms of industrial application. Under examined conversion conditions, the conversion rate of sucrose in pretreated BM remained at a high level exceeding 97.5% within the first eight cycles of repeated batches to produce isomaltulose (Figure 3D). Obviously, after which it decreased slightly from 9 to 11 cycles (>94%) and decreased rapidly beyond 11 cycles (Figure 3D). To avoid disturbing crystal separation of isomaltulose by other sugars and obtain high-purity isomaltulose, the eighth batch was terminated when the conversion rate of sucrose was about 97.5%. The finding was close to that (>90%) by using sucrose substrate isomerized to isomaltulose using the targeted SIase for the first 13 batches (Zhang et al., 2019). Meanwhile, Wu et al. (2016) found that bacterial SIase immobilized onto ε-PL-gelatin remained about 80% conversion rate of sucrose from pure sucrose for the first 156 h. The results stated that immobilized SIase has outstanding operational stability and good reusability using pretreated BM for industrial production of isomaltulose.

### Analysis of Sugar Composition in the Final Reaction Liquid for Isomaltulose Production

**FIGURE 4:**
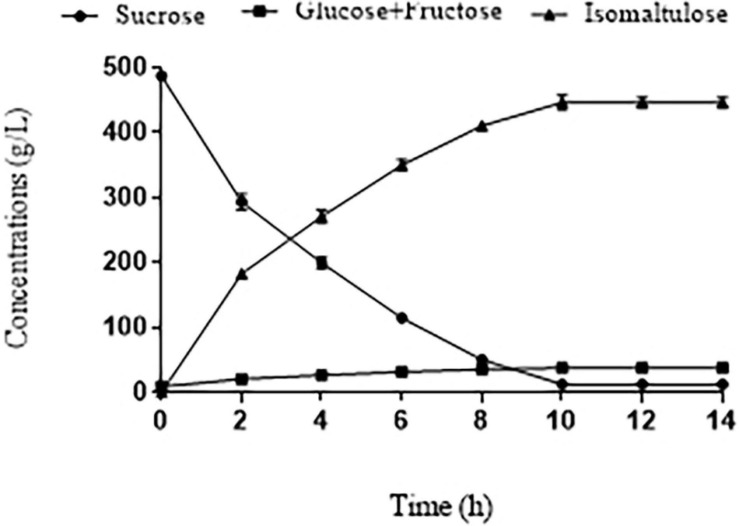
Time course of sucrose conversion in pretreated BM under the best conditions (800 g/L pretreated BM, 40°C, 15 U/g of SIase, pH 5.5) by immobilized SIase in the eighth cycle.

**FIGURE 5:**
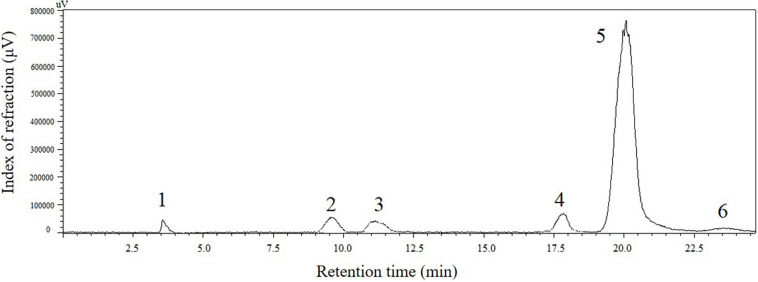
High-performance liquid chromatography (HPLC) profile of products in catalytic liquid of pretreated BM using immobilized SIase in the eighth cycle at 10 h. The numbers from 1 to 6 represent solvent, glucose, fructose, sucrose, isomaltulose, and trehalulose, respectively.

Given the immobilized SIase of the eighth repeated batch still showed a high sucrose conversion rate (97.5%), we conducted the time course of the catalytic reaction. Under the best conditions (800 g/L pretreated BM, 40°C, 15 U SIase/g, pH 5.5) examined above, sucrose along with fast catalytic velocity was reduced to 12.18 g/L at 10 h (Figure 4). Meanwhile, isomaltulose was increasingly accumulated to 446.4 ± 5.5 g/L with the yield of 0.94 ± 0.02 g/g, yet small amounts of glucose and fructose (37.51 g/L), and trehalulose (24.1 g/L) were all slightly elevated due to hydrolysis and isomerization of SIase (Figures 4, 5). The isomaltulose production was improved dramatically than that of an initial condition (Figure 2), which is much higher than those (184.8 g/L, 209.4 g/L, 212.6 g/L) produced by engineered *Y. lipolytica* strains and *Bacillus subtilis* from CM or SM (Wu et al., 2017; Wang et al., 2019b; Zheng et al., 2019). In contrast, the high yield (0.94 ± 0.02 g/g) observed is close to that (0.96 g/g) produced from pure sucrose (Zhang et al., 2018, 2019). These results demonstrated that most of the sucrose in pretreated BM is converted to isomaltulose within 10 h in the eighth batch.

As shown by HPLC analysis in Figure 5, the purity of produced isomaltulose in the final reaction liquid was further identified to be 85.8%, which is lower than (95.5%) pure sucrose (Zhang et al., 2019), but distinctly overs the purity of isomaltulose (64%) derived from SM by *Y. lipolytica* before using other strains to eliminate non-target sugars (Wang et al., 2019b). Interestingly, trehalulose only accounted for 4.6% of the total sugars, similar to the reported result (4%) (Wu and Birch, 2004). It may be attributed to little flux of sucrose to trehalulose supported by SIase and heavy metals, such as Ca^2+^, Ba^2+^, Cu^2+^, Zn^2+^, in BM inhibiting SIase activity (Palmonari et al., 2020; Zhang et al., 2020). Furthermore, dried isomaltulose syrup (content ≥80%), approved as GRAS (No. 681) in 2017 by FDA, can replace sucrose at the same level as sweeteners in foods and beverages (Tian et al., 2019).

Calculated with the conversion rate of the eighth batch, the total yield of isomaltulose per initial mass of pretreated BM was 0.558 g/g, while the total yield of isomaltulose per pure sucrose was 0.96 g/g. Considering the low cost of BM, the yield was desirable. In addition, by repeated enzymatic conversion, immobilized SIase (1 U) could catalyze the synthesis of 0.51 g of isomaltulose. Using fermented SIase with an activity of 49.3 U/mL, SIase of 1 ml taken from the fermentation broth could catalyze the synthesis of 25 g of isomaltulose. Pretreatment can reduce the content of those metal ions in BM, thus increasing the isomaltulose yield and the immobilization of enzyme notably reduced the enzyme demand. These results indicated that the bioprocess using pretreated BM and immobilized SIase is a feasible alternative approach for isomaltulose production.

### Comparison of the Cost for Isomaltulose Production

The cost of sucrose accounts for a substantial part of the total cost of producing isomaltulose based on the sole substitute sucrose or for microbial conversion needing sucrose and other nutrients (Zhang et al., 2019, 2020). The estimation of alternative feedstock cost is indispensable for sustainable industrial production of isomaltulose. The costs of feedstocks were based on the current market prices. As shown in Table 1, high substrate costs for isomaltulose production were evaluated by engineered strains cultivated from sucrose-containing substrates; however, the cost of single BM in this study was observed at 2700 yuan (RMB) per ton isomaltulose based on the whole yield (isomaltulose obtained from the substrate) (Table 1). This reduces the feedstock costs by 25–28.9% and by more than 70%, compared with the cost of other molasses and sucrose as the substrate (Table 1), respectively. More important, the immobilized SIase with high reusability could remarkably reduce the enzyme demand and production cost. The cost of the enzyme can be negligible, in general. Economic analysis revealed that significant cost reduction of isomaltulose production from low-cost BM in this study is reliable for industrial application.

**TABLE 1:** Comparison of the costs from different substrates for isomaltulose production by different engineered food-grade strains and the purity of isomaltulose.

**Strains**	**Substrate cost (yuan/ton isomaltulose)**	**Isomaltulose production (g/L)**	**Isomaltulose proportion (%)**	**References**
*Lactococcus lactis*	Sucrose, 9000	36	<90	Park et al., 2010
*Saccharomyces cerevisiae*	Sucrose, 9000	<4	<10	Lee et al., 2011
*Y. lipolytica*	Sucrose, 8300	572.1	97.8	Zhang et al., 2018
*Bacillus subtilis*	CM, 3800	212.6	<92.4	Wu et al., 2017
*Y. lipolytica*	CM, 3600	161.2	97.4	Wang et al., 2019a
*Y. lipolytica*	SM, 3800	209.4	97.8	Wang et al., 2019b
*Y. lipolytica*	BM, 2700	446.4	85.8	This study

## Conclusion

A two-step process for isomaltulose production from cost-effective BM instead of sucrose was introduced. The H_2_SO_4_ pretreated BM confirmed a more efficient substrate to synthesize isomaltulose than untreated BM, and the immobilized SIase using pretreated BM as a substrate was verified to be the best for isomaltulose production. This process could produce a high concentration level of isomaltulose of 446.4 ± 5.5 g/L with a yield of 0.94 g/g and a purity of 85.8% under the best conditions in the eighth batch, and also remain 97.5% of the sucrose conversion rate. Immobilized SIase used in repeated batch reaction showed good reusability to convert pretreated BM into isomaltulose. It is also economically feasible as shown by economic analysis. This study provides an economical, effective, and promising approach for potential industrial isomaltulose production.

## Data Availability Statement

The original contributions presented in the study are included in the article/supplementary material, further inquiries can be directed to the corresponding authors.

## Author Contributions

Q-QW performed the experiment and wrote the manuscript. J-HH implemented fermentation and revised the manuscript. Z-CM designed the experiment and published the manuscript. All authors contributed to the article and approved the submitted version.

## Conflict of Interest

The authors declare that the research was conducted in the absence of any commercial or financial relationships that could be construed as a potential conflict of interest.
